# Indicators of suboptimal biologic therapy over time in patients with ulcerative colitis and Crohn's disease in the United States

**DOI:** 10.1371/journal.pone.0175099

**Published:** 2017-04-20

**Authors:** Haridarshan Patel, Trevor Lissoos, David T. Rubin

**Affiliations:** 1 Immensity Consulting, Inc., Chicago, Illinois, United States of America; 2 Takeda Pharmaceuticals U.S.A., Inc., Chicago, Illinois, United States of America; 3 Inflammatory Bowel Disease Center University of Chicago Medicine, Chicago, Illinois, United States of America; National Cancer Institute, UNITED STATES

## Abstract

This study assessed the occurrence of indicators for suboptimal biologic therapy among ulcerative colitis (UC) and Crohn’s disease (CD) patients over time in the United States (US). Data from a large US claims database (2005–2013) were used to retrospectively identify patients with diagnosed with either UC or CD who were new biologic users. Indicators of suboptimal biologic therapy included: dose escalation during the maintenance phase, discontinuation of the initial biologic, switch to another biologic within 90 days following the last day of supply of the initial biologic, augmentation with a non-biologic systemic therapy, UC- or CD-related surgery, UC- or CD-related urgent care, and development of fistula (for CD only). Kaplan-Meier analyses were used. A total of 1,699 UC and 4,569 CD patients were included. Among UC patients, 51.1% and 90.9% experienced ≥1 indicator of suboptimal biologic therapy within 6 months and 36 months of biologic therapy initiation, respectively. Among CD patients, 54.3% and 91.4% experienced ≥1 indicator of suboptimal biologic therapy within 6 and 36 months of biologic therapy initiation, respectively. For both UC and CD patients, the most frequent indicators of suboptimal biologic therapy were discontinuation, dose escalation and augmentation. In conclusion, this study found that the occurrence of suboptimal biologic therapy is common among patients with UC and CD, with approximately 90% of patients experiencing at least one indicator of suboptimal biologic therapy within 36 months of biologic treatment initiation.

## Introduction

Ulcerative colitis (UC) and Crohn’s disease (CD) are the two most common clinical forms of inflammatory bowel disease (IBD), a group of chronic inflammatory disorders of the gastrointestinal tract [1]. UC is generally confined to the colon and the rectum, with inflammation and ulcerations primarily affecting the innermost layer or mucosa of the intestine [1, 2]. By contrast, CD can occur anywhere along the gastrointestinal tract and the underlying inflammation can extend to the entire thickness of the intestinal wall [1, 2]. In 2003, the number of people suffering from UC and CD in North America was estimated to be approximately 800,000 and 600,000, respectively [3].

Because UC and CD are characterized by a relapsing and remitting disease course, their clinical management is complex [4, 5]. For patients with mild-to-moderate disease activity, treatment typically begins with so-called conventional medications that include aminosalicylates, corticosteroids, thiopurines, and antibiotics—with regimens optimized and individualized based on disease location, patient preference, and comorbidities [5, 6]. However, conventional treatments do not elicit a response in an estimated 20 to 40% of UC and CD patients [7, 8]. When patients do not respond, lose response, or are intolerant to conventional therapy, clinical guidelines recommend the use of biologic therapy, particularly in the case of moderate-to-severe patients [4, 7, 9–12]. Clinical guidelines also recommend biologic therapy for IBD patients with moderate-to-severe disease who are at higher risk for disease complications. In the United States (US), several biologic therapies are approved for the management of UC and CD, including anti-tumor necrosis factor (anti-TNF) monoclonal antibodies and integrin receptor antagonists [4, 5, 7]. The beneficial effects of biologic therapy on CD and UC symptoms have been demonstrated in multiple studies, with the effects of anti-TNF therapy and vedolizumab extending to mucosal healing and reduced dependence on corticosteroids [4, 7, 13–17].

While the introduction of biologic therapies over the past decade has tremendously advanced the treatment of both UC and CD, not all patients can tolerate or respond equally to biologic therapy. As a result, dose adjustments, augmentation, therapy changes or discontinuation are often required during induction or maintenance therapy [18–20]. For instance, a review study found that up to 46% of CD patients receiving anti-TNFs require dose intensification, while up to 13% discontinue therapy [18]. Recently, another study demonstrated that suboptimal response to biologic therapy—defined by the authors as changes in treatment such as augmentation, treatment switches, dose titration, and discontinuation—is frequent among UC and CD patients and is associated with a substantial economic burden, particularly among CD patients [20]. However, treatment changes were evaluated over a relatively short period of time (2006–2010), the anti-TNF certolizumab pegol was considered only in the case of CD patients, and recently approved biologic therapies such as natalizumab were not included in the analysis. Furthermore, most other previous studies restricted their focus on dose intensification, discontinuation, or loss of response to biologic treatments in either CD or UC and mostly considered one biologic agent at a time [18, 19, 21–23]. A comprehensive evaluation of the rate of treatment changes that are indicative of suboptimal therapy among UC and CD patients in real-world clinical practice is lacking, particularly one that is inclusive of recently approved biologic therapies.

To fill this knowledge gap, the objective of this study was to describe and assess the real-world occurrence of possible indicators of suboptimal biologic therapy—defined by changes in biologic treatment patterns and medical events such as surgery—for UC and CD patients in the US. To this end, we retrospectively analyzed data from a large US claims database over the time period from 2005 to 2013 and included in our analysis a range of biologic treatments.

## Methods

### Data source

Patients were selected from a large US commercial claims database, the Thomson Reuters^®^ MarketScan Commercial Database, for the period from 01/01/2005 to 12/31/2013. The database contains provider and institutional medical claims, pharmacy claims, and the healthcare plan enrollment history of employees, their dependents, and Medicare-eligible retirees with Medicare supplemental plans covered by the health benefit programs of large employers. All US census regions are represented in the database, with a slightly higher representation from the South and North Central (Midwest) regions. The MarketScan database is compliant with the Health Insurance Portability and Accountability Act and thus no institutional review board review was necessary as data do not include any identifiable patient information.

### Sample selection

Patients were included in the study if they met the following inclusion criteria: (1) they had at least two diagnoses of either UC (International Classification of Diseases, Ninth Revision (ICD-9) codes 556.xx) or CD (ICD-9 codes 555.xx) recorded during the entire study period covered by the data; (2) they were at least 18 years old at their first UC or CD diagnosis; (3) they had at least one prescription filled for one of the following biologic therapies: adalimumab, certolizumab pegol, golimumab, infliximab, or natalizumab (vedolizumab was not included, since it had not yet received FDA approval during the period covered by the data); and (4) they had continuous healthcare plan enrollment for at least 12 months before and after the date of the first biologic prescription fill (*index date*). The 12-month period before the index date was included as washout period to identify incident users of biologics.

### Study design and study outcomes

A set of indicators of suboptimal biologic therapy was used—six for UC and seven for CD. Suboptimal biologic therapy was defined as the occurrence of at least one of these indicators, which were defined by the events listed below [20, 24–29]:

1. **Dose escalation**, defined as a dose increase during the maintenance phase resulting in a dose twice as high as the recommended standard maintenance dose for UC and CD [30–34].
2. **Discontinuation**, defined as a gap of at least 60 consecutive days between the last day of supply of the initial biologic therapy for UC or CD (discontinuation date) and either the next prescription fill or the end of continuous healthcare plan enrollment or the end of data availability (i.e., 12/31/2013), whichever occurred first.
3. **Switching**, defined as switching to another biologic treatment for UC or CD within the 90-day period following the last day of supply of the initial biologic therapy. No refills of the previous biologic were allowed within 180 days of initiation of the new biologic.
4. **Augmentation**, defined as the use of a non-biologic systemic therapy for UC or CD (aminosalicylates, immunomodulators/immunosuppressants, oral or injectable corticosteroids, and antibiotics including ciprofloxacin, metronidazole, and rifaximin) as an adjunct along with the initial biologic treatment. At least 28 days of concomitant use of the added non-biologic systemic therapy and the current biologic were required. The non-biologic systemic therapy was also required to have not been used in the 60 days preceding the initiation of the non-biologic systemic therapy. In addition, no refills of any other biologic therapies were allowed after initiation of the non-biologic systemic therapy.
5. **Disease-related surgery**, defined as UC- or CD-related surgery (e.g., colectomy, colostomy, ileostomy, fistula, or abscess repair).
6. **Disease-related urgent care**, defined as the use of UC- or CD-related urgent care services.
7. **Fistula**, defined as the development of fistula (ICD-9 codes 565.1x, 569.81, 599.1x, 619.xx, and 998.5x) in CD patients.

### Statistical analyses

Kaplan-Meier survival analyses were used to estimate the rates of the seven indicators of suboptimal biologic therapy at 6, 12, 24, and 36 months after the first biologic prescription fill. For each indicator, the rates were stratified by disease type (UC or CD). The observation period of patients was censored if either the end of continuous healthcare plan coverage or the end of data availability was reached without the occurrence of any of the indicators of suboptimal biologic therapy. Additionally, in the case of switching, the observation period was censored 90 days after the discontinuation date if no indicator of suboptimal biologic therapy had occurred by that time. Likewise, in the case of dose escalation and augmentation, the observation period was censored at the discontinuation date if no indicator of suboptimal biologic therapy had occurred by that time.

Multivariate Cox proportional hazard regression models were estimated separately for UC and CD patients to identify the factors associated with an increased risk of experiencing any indicator of suboptimal therapy. Results were reported for each factor as adjusted hazard ratios (HR) with their 95% confidence intervals (CI) and p-values. Potential factors were pre-determined based on data availability and clinical relevance. They included age, gender, insurance plan type, and Charlson Comorbidity Index (CCI); for CD patients, they also included indicators of CD severity based on CD location.

## Results

**Fig 1:**
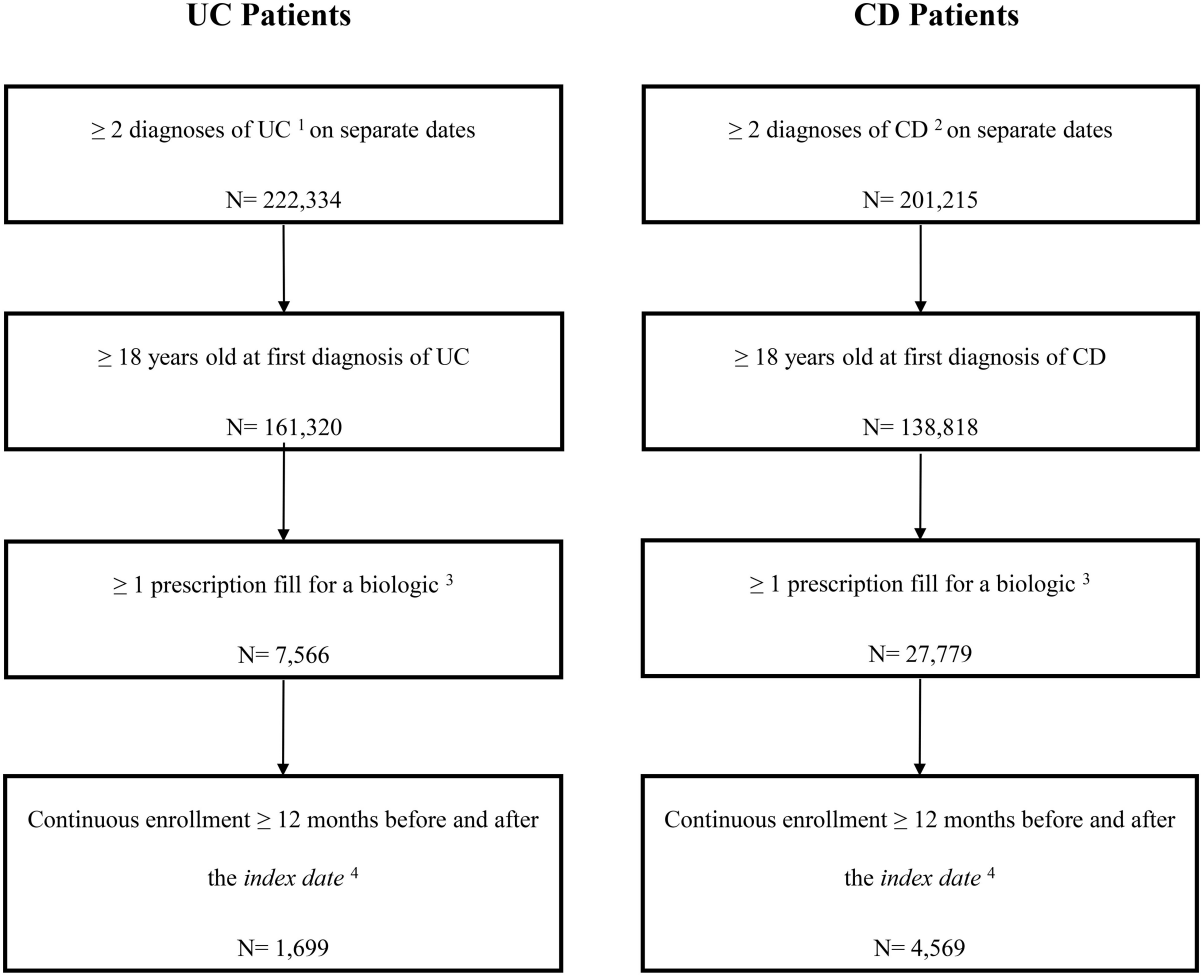
Sample selection. [1] ICD-9 codes 556.xx; [2] ICD-9 codes 555.xx; [3] Biologic agents included adalimumab, certolizumab pegol, golimumab, infliximab, and natalizumab; [4] The index date was defined as the date of the first biologic prescription fill.

A total of 1,699 new biologic users with UC and 4,569 new biologic users with CD were included in the analysis (Fig 1). At the index date, UC patients had a mean age of 47.5 years, and 51.0% of them were female (Table 1). Similarly, CD patients had a mean age of 43.4 years, and 57.9% of them were female (Table 1). For UC patients, the most common biologic therapy used at the index date was infliximab (68.4%) followed by adalimumab (29.3%) and certolizumab (1.5%). For CD patients, the most common biologic therapy used at the index date was adalimumab (48.7%) followed by infliximab (42.5%) and then certolizumab (8.1%).

**Table 1:** Patient characteristics.

Patient Characteristics	UC Patients (N = 1,699)	CD Patients (N = 4,569)
***Age*, *Mean ± SD***	47.5 ± 14.7	43.4 ± 14.7
18–39, n (%)	533 (31.4)	1,899 (41.6)
40–49, n (%)	384 (22.6)	1,044 (22.8)
50–64, n (%)	589 (34.7)	1,312 (28.7)
≥65, n (%)	193 (11.4)	314 (6.9)
***Female*, *n (%)***	867 (51.0)	2,644 (57.9)
***Region*, *n (%)***		
South	691 (40.7)	1,785 (39.1)
North-Central	451 (26.5)	1,401 (30.7)
West	332 (19.5)	763 (16.7)
North-East	225 (13.2)	620 (13.6)
***Insurance Plan Type*, *n (%)***		
Point of Service (POS) or Exclusive Provider Organization (EPO)	211 (12.4)	559 (12.2)
Preferred Provider Organization (PPO)	928 (54.6)	2,579 (56.4)
Health Maintenance Organization (HMO) or POS with capitation	321 (18.9)	831 (18.2)
Consumer-directed Health Plan (CDHP) or High-deductible Health Plan (HDHP)	56 (3.3)	163 (3.6)
Basic or Comprehensive Coverage	183 (10.8)	437 (9.6)
***Biologic Agent Initiated on the Index Date*, *n (%)***		
Adalimumab	498 (29.3)	2,226 (48.7)
Certolizumab	25 (1.5)	369 (8.1)
Infliximab	1,162 (68.4)	1,943 (42.5)
Golimumab	7 (0.4)	7 (0.2)
Natalizumab	7 (0.4)	24 (0.5)
***CD location*, *n (%)***		
Ileum	-	752 (16.5)
Colon	-	486 (10.6)
Ileum/Colon	-	347 (7.6)
Multiple sites	-	333 (7.3)
Not Specified	-	2,651 (58.0)

UC: Ulcerative Colitis; CD: Crohn's Disease

**Fig 2:**
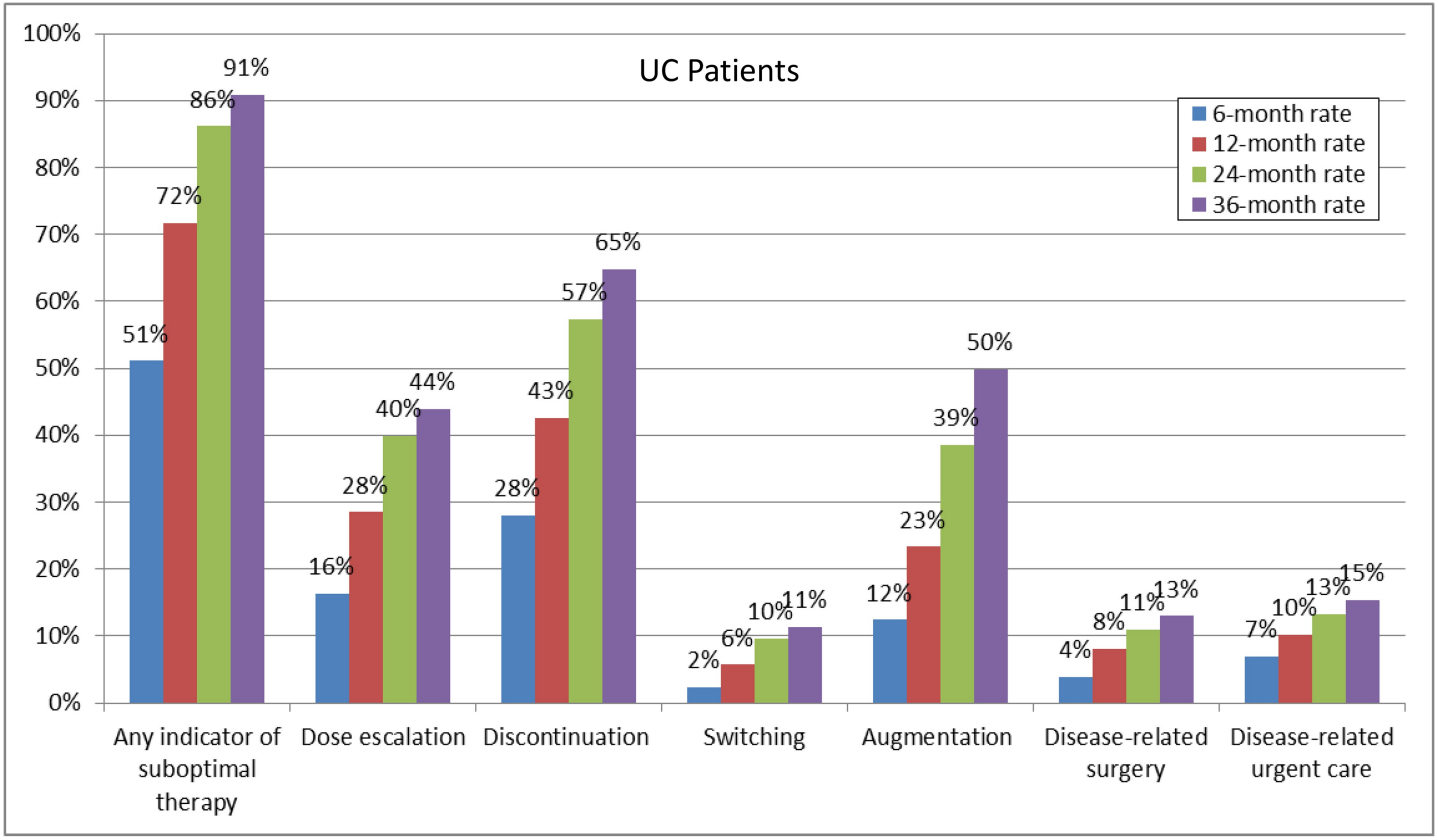
Rates of indicators of suboptimal therapy for UC patients. UC: Ulcerative Colitis.

Among UC patients, 51.1% experienced ≥1 indicator of suboptimal biologic therapy within 6 months of biologic therapy initiation; this rate increased to 90.9% within 36 months of therapy initiation (Fig 2). The most frequent indicators of suboptimal biologic therapy were discontinuation of index biologic therapy (ranging from 28.1% to 64.8% from 6 to 36 months), biologic dose escalation (ranging from 16.3% to 44.0% from 6 to 36 months), and augmentation with a non-biologic systemic therapy (ranging from 12.4% to 49.9% from 6 to 36 months).

**Fig 3:**
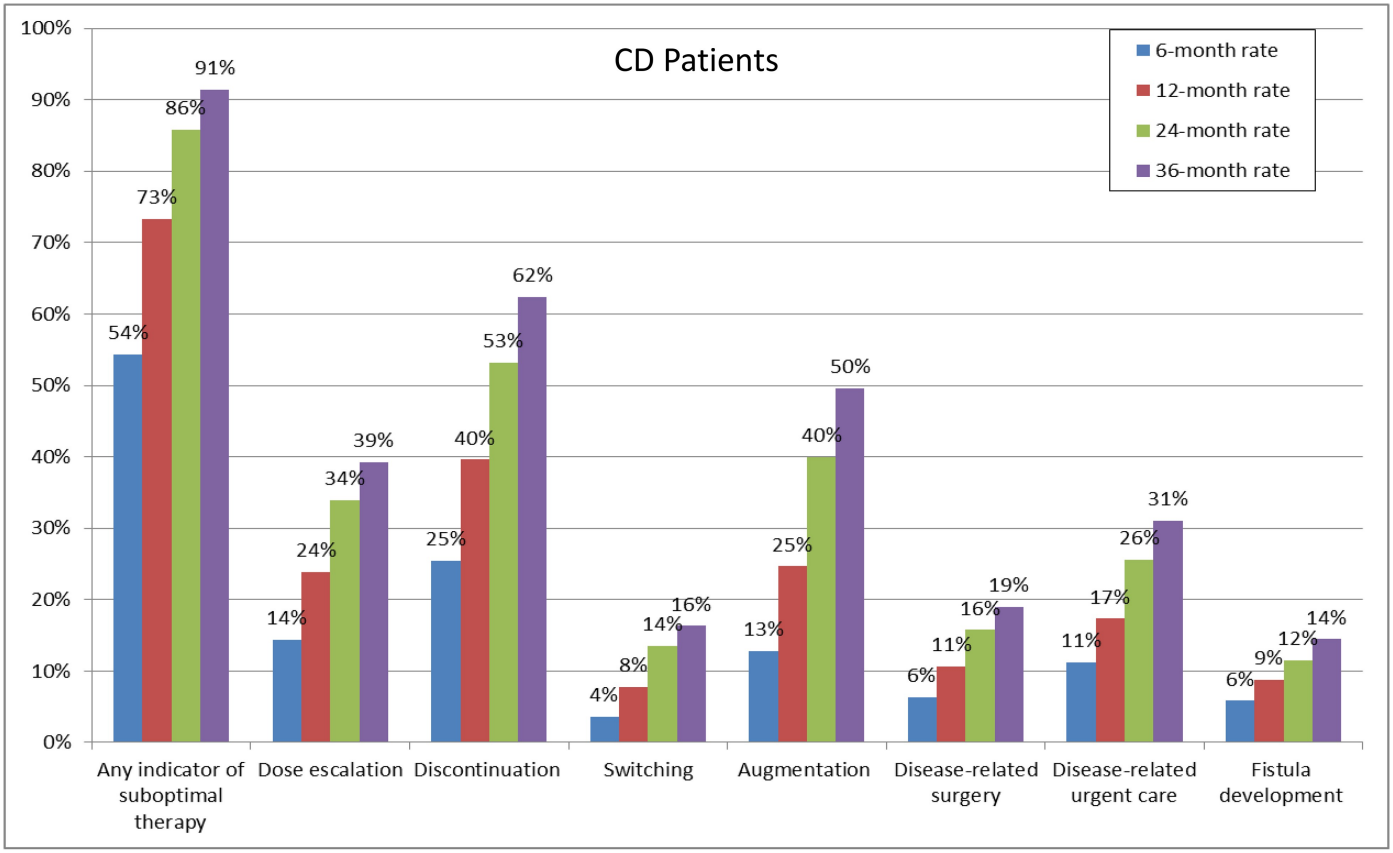
Rates of indicators of suboptimal therapy for CD patients. CD: Crohn's Disease.

Among CD patients, 54.3% and 91.4% experienced ≥1 indicator of suboptimal biologic therapy within 6 and 36 months, respectively (Fig 3). The most frequent indicators of suboptimal biologic therapy were discontinuation of index biologic therapy (ranging from 25.5% to 62.4% from 6 to 36 months), biologic dose escalation (ranging from 14.4% to 39.3% from 6 to 36 months), and augmentation with a non-biologic systemic therapy (ranging from 12.8% to 49.6% from 6 to 36 months).

In both UC and CD, females and patients with a greater comorbidity burden (higher CCI) were significantly more likely to experience an indicator of suboptimal biologic therapy (Table 2).

**Table 2:** Factors associated with indicators of suboptimal therapy for UC and CD patients.

	UC Patients (N = 1,699)	CD Patients (N = 4,569)
	HR (95% CI)	HR (95% CI)
***Age***		
18–39	*Reference*	*Reference*
40–49	1.08 (0.94; 1.25)	0.98 (0.91; 1.07)
50–64	1.02 (0.89; 1.16)	0.94 (0.87; 1.01)
≥65	1.13 (0.92; 1.40)	0.98 (0.84; 1.14)
***Female*** (*Reference = Male*)	*1*.*12 (1*.*01; 1*.24)*	*1*.*19 (1*.*12; 1*.27) *
***Insurance Plan Type***		
Point of Service (POS) or Exclusive Provider Organization (EPO)	*Reference*	*Reference*
Preferred Provider Organization (PPO)	1.00 (0.85; 1.18)	0.95 (0.86; 1.04)
Health Maintenance Organization (HMO) or POS with capitation	1.11 (0.92; 1.33)	1.05 (0.93; 1.17)
Consumer-directed Health Plan (CDHP) or High-deductible Health Plan (HDHP)	1.13 (0.81; 1.56)	0.97 (0.81; 1.18)
Basic or Comprehensive Coverage	0.84 (0.66; 1.06)	1.00 (0.86; 1.15)
***Region*** *(reference = South)*		
North-Central	1.03 (0.91; 1.18)	0.90 (0.84; 0.97)
North-East	0.98 (0.83; 1.16)	0.97 (0.88; 1.07)
West	0.98 (0.85; 1.13)	0.94 (0.86; 1.03)
***CCI***	*1*.*05 (1*.*01; 1*.10)*	*1*.*06 (1*.*02; 1*.09)*
***CD location*** *(reference = ileum)*		
Colon	NA	0.95 (0.84; 1.07)
Ileum/Colon	NA	0.95 (0.83; 1.09)
Multiple sites	NA	0.92 (0.80; 1.06)
Not Specified	NA	0.99 (0.90; 1.07)

UC: Ulcerative Colitis; CD: Crohn's Disease; HR: Hazard Ratio; CI: Confidence Interval; CCI: Charlson Comorbidity Index

## Discussion

Biologic therapies are being increasingly used for the management of UC and CD and optimizing these therapies to maximize primary response and minimize loss of response is essential to their proper use. Despite the clear importance of optimizing biologic therapies, the prevalence of suboptimal biologic therapy has been poorly characterized in the existing literature. To address this issue, this large-scale, real-world study evaluated the occurrence of a wide range of indicators of suboptimal biologic therapy among UC and CD patients in the US. Importantly, changes in the occurrence of such indicators were evaluated over time, from 6 months to 36 months after biologic treatment initiation.

Our results showed that more than half of UC and CD patients experienced at least one indicator of suboptimal biologic therapy within 6 months of biologic treatment initiation. The proportion of both UC and CD patients with at least one indicator of suboptimal biologic therapy steadily increased over time, exceeding 90% after 36 months.

The most common indicators of suboptimal biologic therapy were found to be treatment discontinuation, dose escalation, and augmentation. Remarkably, within 36 months of biologic therapy initiation, more than 60% of both UC and CD patients discontinued treatment. In addition, approximately 50% and 40% of both UC and CD patients underwent augmentation and experienced dose escalation, respectively.

Our results are largely in agreement with a previous study of administrative claims data investigating therapy changes and suboptimal treatment among incident UC and CD patients 12 months after first-line pharmacological therapy initiation in monotherapy [20]. In that study, the 12-month prevalence of biologic dose escalation was estimated at 27% and 28% for UC and CD patients, respectively, in line with our estimates. As in the present study, discontinuation and augmentation were also found to be common. However, since the prevalence of these two indicators in both patient populations was reported separately for only two biologic therapies—adalimumab and infliximab—a direct comparison involving discontinuation and augmentation rates between the two studies is challenging. Different study populations further complicate direct comparisons.

Nevertheless, the fact that both of these studies found that indicators of suboptimal therapy are frequent among UC and CD patients receiving biologic therapies in real-world clinical practice highlights the substantial unmet need that exists in the management of UC and CD. As suboptimal therapy affects clinical outcomes and may affect the quality of life of patients suffering from UC and CD, it is important that physicians and patients have a realistic understanding of how biologic therapies require augmentation or changes within 36 months of initiation. A better understanding of the factors underlying the high rates of suboptimal biologic therapy observed here among UC and CD patients will be instrumental in the development of long-term, stable treatment strategies and thus in the reduction of salvage therapy rates.

A major strength of the current study is the inclusion of more recently approved biologic therapies, which were not captured in the previous claims data study [20]. Since only limited information is available on the prevalence of suboptimal therapy among UC and CD patients treated with biologic therapies, this study provides a much-needed and updated measure of suboptimal biologic therapy in US clinical practice. However, it should be acknowledged that our study sample might have included patients who did not receive optimal anti-TNF dosing [35, 36]. As previous studies have shown, in fact, only an anti-TNF concentration >3 μg/mL is associated with clinical response and lower disease activity [37]. Furthermore, multiple factors might affect the pharmacokinetics of anti-TNF levels, including albumin, weight, gender, inflammation, administration route, and immunogenicity [37]. Notwithstanding the multiplicity of factors that might influence response to biologic therapy, no single marker has been identified so far as a reliable predictor of response or suboptimal therapy in UC and CD [38, 39]. In this study, in the absence of routine therapeutic drug monitoring and predictive indicators of response to biologic therapy, treatment changes were used as proxies for loss of response.

The study findings should be interpreted in light of some limitations inherent to claims data. First, claims data may be subject to medical coding errors or inaccuracies, which we attempted to minimize in our methods. Second, since our study population was commercially insured, our results might not be generalizable to US patients who are enrolled in Medicare and Medicaid or have no or limited healthcare coverage; similarly, our results might not be generalizable to UC and CD patients outside the US. It should also be noted that our study population was limited to first-time users of biologic therapies. Third, claims for medications or medical services that are fully reimbursed by Medicare do not appear in the database. Fourth, since our definition of indicators of suboptimal therapy was based on events indirectly indicative of suboptimal therapy and clinical measures of treatment response are not available in claims data, objective confirmation of suboptimal therapy could not be established. Therapy augmentation and dose escalation may not always be indicative of suboptimal therapy; indeed, they could also be prescribed for proactive therapeutic drug monitoring or may indicate that disease symptoms are not adequately controlled. Regardless of the type of therapy used, because of the natural history of the disease, patients may experience episodes of more aggressive disease. Finally, clinical characteristics that might influence treatment selection and duration, such as disease severity, are not available in claims databases and thus could not be assessed.

## Conclusions

The results of this real-world study showed that more than half of UC and CD patients experienced at least one indicator of suboptimal biologic therapy within 6 months of initiation of their first biologic. This proportion of patients increased over time, up to approximately 90% within 36 months of first biologic treatment initiation. These findings have considerable importance for improving the management of UC and CD patients.
